# Incidental aortic valve papillary fibroelastoma diagnosed by transesophageal echocardiography in a patient undergoing coronary artery bypass surgery: a case report

**DOI:** 10.1093/jscr/rjae679

**Published:** 2024-10-29

**Authors:** Nael Al-Sarraf, Adel Maher, Yuldash Agzamov, Mohammed Hasan, Ali Alhumaidan

**Affiliations:** Department of Cardiac Surgery, Dabbous Cardiac Center, Adan Hospital, Kuwait City, Kuwait; Department of Cardiac Surgery, Dabbous Cardiac Center, Adan Hospital, Kuwait City, Kuwait; Department of Cardiac Surgery, Dabbous Cardiac Center, Adan Hospital, Kuwait City, Kuwait; Department of Cardiology, Dabbous Cardiac Center, Adan Hospital, Kuwait City, Kuwait; Department of Cardiology, Dabbous Cardiac Center, Adan Hospital, Kuwait City, Kuwait

**Keywords:** papillary fibroelastoma, cardiac tumor, aortic valve, echocardiography

## Abstract

Papillary fibroelastoma of aortic valve is a rare benign tumor that can present with symptoms of obstruction or embolization and can be asymptomatic. The main stay of diagnosis is echocardiography. The size of the tumor affects the sensitivity of transthoracic echocardiography which can miss small size tumors. The treatment is surgical resection. Here, we report a case of papillary fibroelastoma of aortic valve that was detected intraoperatively by transesophageal echocardiography and was missed by transthoracic echocardiography in a patient undergoing coronary artery surgery. The tumor was resected successfully with preservation of aortic valve with no complication. This case highlights importance of intraoperative transesophageal echocardiography in making the diagnosis.

## Introduction

Papillary fibroelastoma (PFE) is a rare benign tumor that can vary in size and symptomatology. The mainstay of diagnosis is echocardiography with higher sensitivity of transesophageal echocardiography (TEE) over transthoracic echocardiography (TTE) in tumor detection. Here, we report a rare case of PFE of aortic valve in a patient scheduled to undergo coronary artery bypass graft (CABG) surgery where TEE made the diagnosis that was missed by preoperative TTE.

## Case report

**Figure 1:**
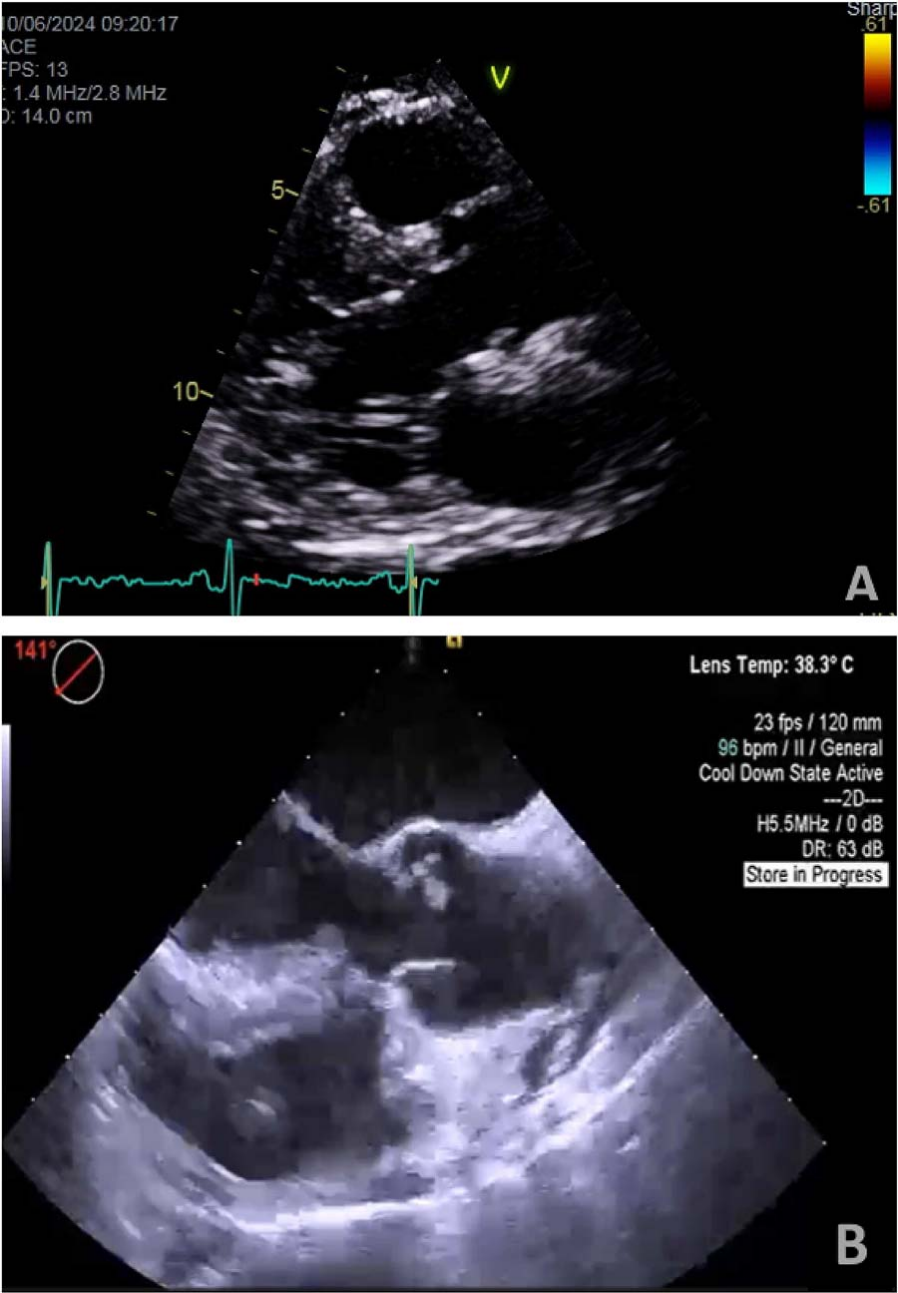
(A) Preoperative transthoracic echo (Para sternal long axis view) showing clear aortic valve cusps with no mass. (B) Intraoperative transesophageal echo (midesophageal long-axis view at 140°–150°) showing mass attached to ventricular surface of NCC of aortic valve.

**Figure 2:**
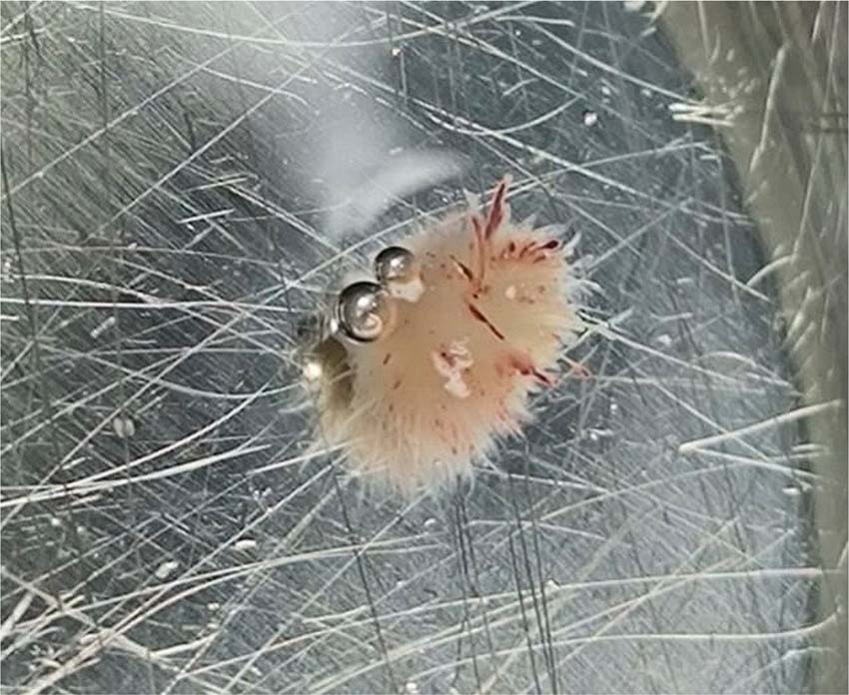
PFE of aortic valve following excision.

**Figure 3:**
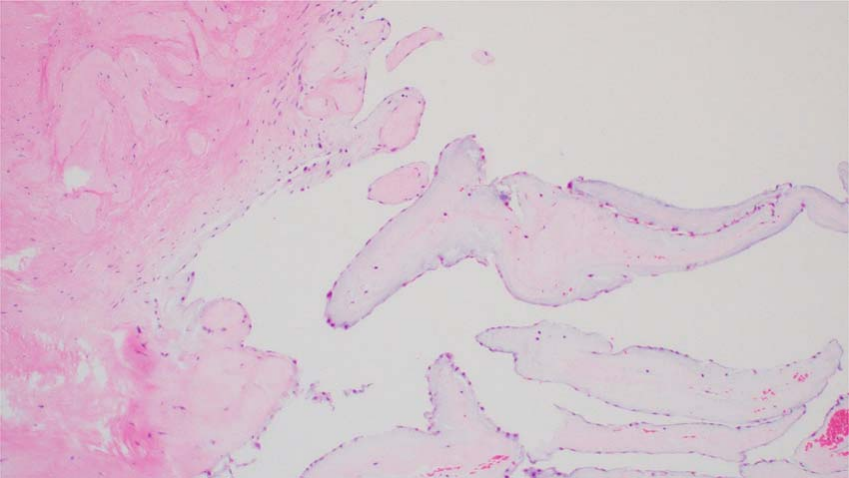
Hematoxylin and Eosin (H&E) stain of the tumor showing multiple branching fronds of paucicellular fibroelastic tissue lined by a single endocardium. Magnification power ×100.

A 55-year-old man with hypertension and hyperlipidemia presented to our hospital with non-ST-segment myocardial infarction (NSTEMI). He was a smoker with no family history of heart disease. Patient was having class III angina for the previous 6 months but never sought medical help. He had no shortness of breath and no history of previous cerebrovascular accident (CVA). The patient underwent coronary angiogram that showed triple vessel disease with chronically occluded right coronary artery. Preoperative TTE showed left ventricular ejection fraction of 50% with mild mitral valve regurgitation and no aortic valve disease (Fig. 1A). Chest radiography was normal and his preoperative blood tests including C-reactive protein, Erythrocyte sedimentation rate (ESR), and complete blood count were normal. Blood cultures were negative. Patient underwent CABG during the same hospitalization due to his recurrent chest pain. Intraoperative TEE (Fig. 1B) showed a mass in the undersurface (ventricular side) of the non-coronary cusp (NCC) of aortic valve with normal valve function (tri leaflet valve). The differential diagnosis at that time included vegetation, calcification, or tumor. Median sternotomy was performed with ascending aorta and right atrial venous cannulation. CABG was performed with left internal mammary artery anastomosis to left anterior descending artery and reverse saphenous vein graft anastomosis to obtuse marginal artery. The aortic valve was exposed through transverse aortotomy and the mass was visualized in the ventricular side of NCC of aortic valve measuring 6 × 5 mm. It was white in color, rounded with some fronds (Fig. 2). The aortic valve was tri-leaflet valve with some calcifications noted in the other two cusps. The mass was then shaved off the valve and sent to histopathology. The leaflet was intact, and the aortic valve was tested and was normal with no regurgitation. Patient tolerated the procedure well and was weaned from cardiopulmonary bypass with small dose of Dobutamine. TEE at the end of operation showed well-functioning aortic valve with no aortic regurgitation. Patient was extubated 7 hours postoperatively and he had uncomplicated postoperative recovery and was discharged home 1 week postoperatively on dual antiplatelet therapy (Aspirin and Clopidegrol). Pathology report of the aortic mass (Fig. 3) showed 0.6 × 0.5 × 0.4 cm PFE (microscopic section showed multiple branching fronds and paucicellular avascular fibro elastic tissue lined by a single layer of endocardium). Patient remained well and asymptomatic at 3 months postoperatively.

## Discussion

PFEs are rare and slow-growing avascular benign tumors with sizes ranging from 2 to 70 mm. They are classically characterized by multiple papillary fronds which give a sea anemone-like appearance. Histologically they are comprised of a single layer of endocardial cells covering the papillary surface, matrix consisting of longitudinally oriented collagen with irregular elastic fibers, proteoglycans and spindle cells that resemble smooth muscle cells or fibroblasts [1]. Commonest location reported is in aortic valve (involving right or non-coronary cusps) in 44.5% of cases and more commonly are on the superior surface (aortic side) than inferior surface of aortic valve (ventricular side) [2]. Majority are single tumors and 44% of them are mobile with a stalk [2]. They are predominantly diagnosed in men at the age of 60 years old with half of patients being asymptomatic. Symptoms reported include dyspnea, CVA, angina, and syncope [2–5]. TTE has a diagnostic accuracy of 85% and classically PFE are small <15 mm round echo-dense and pedunculated mass with high independent motion. Its characteristic shimmery appearance correlated with filiform projections. However, detection of small PFE < 5 mm requires higher resolution than that offered by TTE (as in our case which was missed by TTE and picked up by TEE) [1]. TEE is reported to have higher sensitivity for tumors <2 mm than TTE (77 vs 62%, respectively) [2]. The reasons why TTE may fail to diagnose tumors include the following: when tumor was masked by an associated lesion, too small to be seen, examination was not done carefully with sufficient index of suspicion, and if there were no significant characteristics to differentiate tumor from the degenerative valve disease [2]. Surgical resection remains the treatment of choice in all symptomatic patients or asymptomatic patients if tumor size >1 cm or has increased mobility or undergoing another cardiac procedure as in our case.

## Conclusion

The use of intraoperative TEE is of a paramount importance in detection of PFE in patients undergoing cardiac surgery especially in small size tumors that can be otherwise missed by preoperative TTE.
